# Effects of Dithiothreitol on Fertilization and Early Development in Sea Urchin

**DOI:** 10.3390/cells10123573

**Published:** 2021-12-17

**Authors:** Nunzia Limatola, Jong Tai Chun, Sawsen Cherraben, Jean-Louis Schmitt, Jean-Marie Lehn, Luigia Santella

**Affiliations:** 1 Department of Research Infrastructures for Marine Biological Resources, Stazione Zoologica Anton Dohrn, 80121 Napoli, Italy; nunzia.limatola@szn.it; 2 Department of Biology and Evolution of Marine Organisms, Stazione Zoologica Anton Dohrn, 80121 Napoli, Italy; 3 Laboratory of Supramolecular Chemistry, Institut de Science et d’Ingénierie Supramoléculaires ISIS—Université de Strasbourg, 8 allée Gaspard Monge, 67000 Strasbourg, France; sawsen.cherraben@sorbonne-universite.fr (S.C.); jlschmitt@unistra.fr (J.-L.S.); lehn@unistra.fr (J.-M.L.)

**Keywords:** vitelline layer, fertilization, sea urchin eggs, plasticity, Ca^2+^ signaling, actin, DTT, TCEP, BPA-C8-Cy3, electron microscopy

## Abstract

The vitelline layer (VL) of a sea urchin egg is an intricate meshwork of glycoproteins that intimately ensheathes the plasma membrane. During fertilization, the VL plays important roles. Firstly, the receptors for sperm reside on the VL. Secondly, following cortical granule exocytosis, the VL is elevated and transformed into the fertilization envelope (FE), owing to the assembly and crosslinking of the extruded materials. As these two crucial stages involve the VL, its alteration was expected to affect the fertilization process. In the present study, we addressed this question by mildly treating the eggs with a reducing agent, dithiothreitol (DTT). A brief pretreatment with DTT resulted in partial disruption of the VL, as judged by electron microscopy and by a novel fluorescent polyamine probe that selectively labelled the VL. The DTT-pretreated eggs did not elevate the FE but were mostly monospermic at fertilization. These eggs also manifested certain anomalies at fertilization: (i) compromised Ca^2+^ signaling, (ii) blocked translocation of cortical actin filaments, and (iii) impaired cleavage. Some of these phenotypic changes were reversed by restoring the DTT-exposed eggs in normal seawater prior to fertilization. Our findings suggest that the FE is not the decisive factor preventing polyspermy and that the integrity of the VL is nonetheless crucial to the egg’s fertilization response.

## 1. Introduction

For a cell like the sea urchin egg, the presence of a well-developed extracellular matrix is of particular importance. The extracellular matrix not only protects the cell by covering its surface but also plays multiple roles in cell−cell communication. For example, sperm-activating peptides released from the jelly coat of sea urchin eggs serve as a chemoattractant for the sperm [1,2,3]. On the other hand, the vitelline layer (VL) of a sea urchin egg, which intimately covers the plasma membrane, has long been recognized as the primary subcellular site of sperm attachment during fertilization [4,5,6]. Indeed, bindin, a protein isolated from the acrosomal process of sea urchin sperm, was shown to mediate species-specific attachment to the egg’s VL, and the egg’s receptor for sperm or bindin has also been isolated from the VL [7,8,9,10,11,12,13,14]. Nevertheless, it is also true that the VL has often been removed or circumvented in order to facilitate the experimental procedure [15,16]. It has been generally assumed that such a practice does not affect the fertilization process and embryonic development, while its effect on the cytological and biochemical changes characterizing egg activation has not been addressed sufficiently.

The fertilization process comprises a series of sequential events such as sperm chemotaxis and activation, sperm adhesion and fusion with the oolemma, egg penetration, and finally the fusion of the male and female pronuclei [11,17,18]. One of the hallmarks of fertilization in virtually all animal species is the increase of Ca^2+^ inside the eggs [19,20,21,22,23]. In sea urchin, a fertilized egg exhibits two modes of Ca^2+^ increase: immediate Ca^2+^ influx and slow propagation of a Ca^2+^ wave (see below) [20,24]. In parallel to this, some cortical actin filaments translocate to the inner cytoplasm, while others elongate the microvilli in the perivitelline space [25,26,27,28,29]. These progressive changes of the Ca^2+^ signals and the reorganization of the cortical actin cytoskeleton are believed to play diverse roles in egg activation and in early development [30,31,32,33]. 

The mobilization of intracellular Ca^2+^ and actin filaments at fertilization should be under tight control because their deregulation is often accompanied by developmental problems [18,27,28,30,34]. The present study is on the extension of a series of our previous studies on egg quality and the roles of the cortical actin cytoskeleton in fertilization [18,27,28,29,30,31,32,33]. This time, we focused on the effect of reducing agents. As aforementioned, DTT has been utilized to “safely” remove the extracellular matrix, such as the egg VL, which has facilitated a number of experiments. However, DTT may affect a host of proteins involved in fertilization and egg activation. Indeed, the sea urchin egg’s receptor itself for sperm binding at the VL is a multisubunit complex linked by disulfide bonds [35,36] that are sensitive to DTT. Furthermore, in view of the fact that the extracellular matrix is an important part of the cell surface that constantly receives chemical and mechanical signals from the external space [37,38], it is conceivable that even a modest alteration of the structure may influence the aforementioned biological processes related to fertilization and egg activation. 

In this context, our specific question here is whether and how the reducing condition involving DTT, which is mild enough not to remove the VL of the eggs, would affect the cell physiology of sea urchin eggs. It was found that treatment of sea urchin eggs (*Lytechinus pictus*) with DTT results in the removal of VL in a time- and pH-dependent manner, without affecting the fertilization response and embryo development [15]. In that study, 10 mM DTT (7–10 min) was effective at removing the VL only above a certain pH, such as 9.2. In our experiment, the NSW containing 10 mM DTT exhibited pH 7.57, which is within the range that would not effect the removal of the VL within 10 min. After examining how the incubation condition (NSW with 10 mM DTT, 10 min) alters the VL and other ultrastructures of *Paracentrotus lividus* eggs through the use of electron microscopy, we assessed the effect of the same reducing conditions on the cytoskeletal properties of the eggs, as well as on its physiological responses to fertilizing sperm. In addition, we tested whether the physiological effect of DTT could be reversed by restoring DTT-exposed eggs in the normal seawater prior to fertilization.

## 2. Materials and Methods

### 2.1. Gametes Collection, Fertilization Procedure, and Embryos Observation

Sea urchins (*Paracentrotus lividus*) were collected from October to May in the Gulf of Naples and were maintained at 16 °C in circulating seawater. Spawning was induced by intracoelomic injection of 0.5 M KCl, and the resulting eggs were collected in natural seawater (NSW) filtered with a Millipore membrane of 0.2 μm pore size (Nalgene vacuum filtration system, Thermo Fisher Scientific, Rochester, NY, USA). For fertilization, dry sperm collected by pipetting on the male animal’s body were kept at 4 °C and diluted in NSW only a few minutes before fertilization. The final sperm concentration for egg insemination was 1.84 × 10^6^ cells/mL. The subsequent embryonic development was observed with a Leica DMI6000 B inverted microscope. By default, the number of zygotes examined for each experiment was 100, and three independent experiments were conducted with different batches.

### 2.2. Visualization of the Egg-Incorporated Sperm

*P. lividus* sperm were prepared afresh and stained with 5 µM Hoechst-33342 (Sigma-Aldrich, St. Louis, MO, USA) for 30 s prior to fertilization. Diluted sperm (10 μL) were added to the media containing the eggs (1 mL). The number of egg-integrated sperm was counted 5 min after insemination by epifluorescence microscopy with a cooled CCD (charge-coupled device) camera (MicroMax, Princeton Instruments, Inc., Trenton, NJ, USA) mounted on a Zeiss Axiovert 200 inverted microscope (Carl Zeiss AG, Oberkochen, Germany) with a Plan-Neofluar 40×/0.75 objective and a UV laser. The Hoechst-33342 solution used in this condition was able to visualize both male and female pronuclei in the zygote when viewed in a Leica TCS SP8X confocal laser scanning microscope equipped with a Diode 405 laser and hybrid detectors (Leica Microsystem, Wetzlar, Germany). The number of fertilized eggs being examined (n) and the number of the independently repeated experiments (N) for each condition are specified in Table 1 and Table 2.

### 2.3. Scanning Electron Microscopy (SEM)

*P. lividus* eggs from three animals were fixed directly in NSW containing 0.5% glutaraldehyde (pH 8.1) for 1 h at room temperature, and post-fixed with 1% osmium tetroxide for an additional hour. The specimens obtained before and after fertilization (at different time points) were dehydrated in increasing concentrations of ethanol and were subjected to critical point drying (LEICA EM CP300). The samples were then coated with a thin layer of gold using a LEICA ACE200 sputter coater, and at least five different eggs for each condition were observed with a JEOL 6700F scanning electron microscope (Akishima, Tokyo, Japan).

### 2.4. Transmission Electron Microscopy (TEM)

Eggs from three animals were fixed before and at different time points after fertilization, directly in NSW containing 0.5% glutaraldehyde (pH 8.1) for 1 h at room temperature, and were post-fixed with 1% osmium tetroxide and 0.8% K_3_Fe(CN)_6_ for another hour at 4 °C. After washing in NSW for 10 min twice, the samples were rinsed in distilled water for 10 min twice and subsequently treated with 0.15% tannic acid for 1 min at room temperature. After extensive rinsing in distilled water (three times, 10 min each), the specimens were dehydrated in increasing concentrations of ethanol followed by propylene oxide for embedding in Epon 812. Ultrathin sections (70 nm) were stained with UAR-EMS (Uranyl Acetate Replacement Stain, Electron Microscope Sciences, Hatfield, PA, USA) for 30 min, and with 0.3% lead citrate for 30 s. Then, at least five different eggs for each condition were observed with a transmission electron microscope (Zeiss LEO 912 AB).

### 2.5. Chemicals and Reagents

DL-Dithiothreitol (DTT) (Sigma-Aldrich, St. Louis, MO, USA) and Tris-(2-carboxyethyl)phosphine hydrochloride (TCEP) (ThermoFisher, Waltham, MA, USA) were dissolved in distilled water (DW) and used for bath incubation at the concentrations specified in the text. Hoechst-33342 and all other unspecified materials were purchased from Sigma Aldrich. BPA-C8-Cy3 was synthesized by following the procedure specified in the Supplementary Material File S1.

### 2.6. Microinjection, Ca^2+^ Imaging, and Confocal Microscopy

Intact eggs were microinjected using an air pressure transjector (Eppendorf FemtoJet, Hamburg, Germany), as previously described [39]. To monitor changes in the intracellular Ca^2+^ levels at fertilization, 500 µM Calcium Green 488 conjugated with 10 kDa dextran was mixed with 35 µM Rhodamine Red (Molecular Probes, Eugene, OR, USA) in the injection buffer (10 mM Hepes, 0.1 M potassium aspartate, pH 7.0) and microinjected into the eggs before insemination. The fluorescence signals of the cytosolic Ca^2+^ increases were captured with a cooled CCD camera (Micro-Max, Princeton Instruments) mounted on a Zeiss Axiovert 200 microscope with a Plan-Neofluar 40×/0.75 objective at about 3 s intervals, and the data were analyzed with MetaMorph (Universal Imaging Corporation, Molecular Devices, LLC, San Jose, CA, USA). Following the formula F_rel_ = [F − F_0_]/F_0_, where F represents the average fluorescence level of the entire egg and F_0_ the baseline fluorescence, the overall Ca^2+^ signals were quantified for each moment and F_rel_ was expressed as RFU (relative fluorescence unit) for plotting the Ca^2+^ trajectories. Applying the formula F_inst_ = [F_t_ − F_(t−1)_]/F_(t−1)_, the instantaneous increments of the Ca^2+^ level was analyzed to locate the specific area of momentary Ca^2+^ increase. The values of Ca^2+^ signals were obtained from four independent experiments (N), and the number of the eggs (n) being analyzed for each condition is specified in the Results.

To visualize F-actin in living eggs, 10 µM (pipette concentration in methanol) of AlexaFluor568-phalloidin (Molecular Probes) was microinjected into the eggs in three independent experiments utilizing as many female animals. To visualize the plasma membrane and the extracellular layers, eggs from two different females were incubated with 5 µM FM 1-43 (ThermoFisher Scientific) or 50 µM of a branched fluorescent polyamine (BPA-C8-Cy3) for 10 min in two independent experiments. Both probes were dissolved in distilled water. The eggs treated with the fluorescent probes were observed with a Leica TCS SP8X confocal laser scanning microscope equipped with a white light laser and hybrid detectors (Leica Microsystem, Wetzlar, Germany). The number of eggs examined for each condition is specified in the Results.

### 2.7. Statistical Analysis

The numerical MetaMorph data were compiled and analyzed with Excel (Microsoft Office 2010) and reported as mean ± standard deviation (SD in all cases in this manuscript. Oneway ANOVA and U-test were performed through Prism 8.0 (GraphPad Software), and *p* < 0.05 was considered to be statistically significant. For ANOVA results showing *p* < 0.05, statistical significance of the difference between the two groups was assessed by Tukey’s post hoc tests. The two groups of data showing significant differences from each other were marked with brackets and symbols indicating the *p* values. The pairwise comparison that produced insignificant *p* values (>0.05) were not mentioned for the sake of simplicity in the description.

### 2.8. Ethics Statement

Sea urchins *P. lividus* used for the present study were collected according to the Italian legislation (DPR 1639/68, 19 September 1980 and confirmed on 1 October 2000). All the experimental procedures were carried out in accordance with the guidelines of the European Union (Directive 609/86).

## 3. Results

### 3.1. DTT Induces Ultrastructural Changes on the Surface of Unfertilized Sea Urchin Eggs

**Figure 1:**
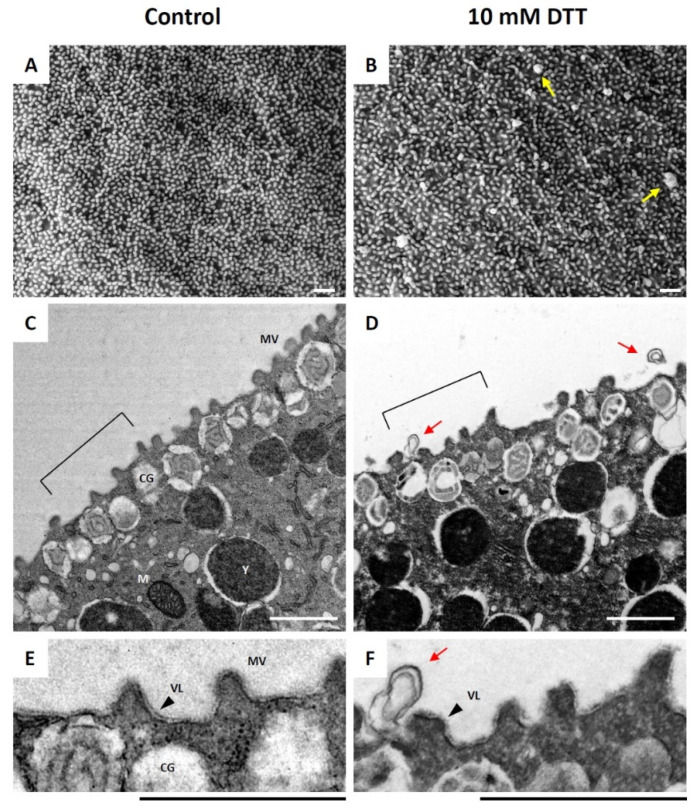
Ultrastructure of unfertilized sea urchin eggs at the surface after brief treatment with dithiothreitol. *P. lividus* eggs were exposed to 10 mM dithiothreitol (DTT) in seawater for 10 min and were subjected to analyses by electron microscopy, following the procedures described in Materials and Methods. (**A**,**B**) Images obtained by SEM. (**C**,**D**) Images obtained by TEM. Note that, after the DTT treatment, the shape of the individual microvilli (MV) became irregular and slightly thicker, and that numerous blebs were formed on the egg surface (yellow and red arrows in **B**,**D**,**F**). (**E**,**F**) For closer examination, the areas of the TEM images marked by brackets in panels (**C**,**D**) are enlarged. Scale bars, 1 μm. CG—cortical granules; MV—microvilli; M—mitochondria; Y—yolk granules; VL—vitelline layer.

As shown in Figure 1, the pretreatment of unfertilized *P. lividus* eggs had subtle but evident effects on the egg surface. Although the topography of the egg surface, which is characterized by the presence of a myriad of microvilli, was not drastically changed by DTT treatment, the individual microvilli became more irregular in shape and occasionally thicker (Figure 1B). In addition, the SEM and TEM images revealed the formation of numerous blebs as big as 0.5 μm on the surface of the eggs treated with DTT (Figure 1B,D arrows), which is in line with earlier findings with *S. purpuratus* eggs treated in the same condition [40]. These blebs were previously interpreted as expelled cortical granules [40]. More importantly, the enlarged view of the TEM images revealed that the continuous contour of the VL, which was evident in the control eggs, was intermittently interrupted or fuzzy in the eggs treated with 10 mM DTT for 10 min (Figure 1E,F). Thus, it appears that the given condition of the egg preincubation with DTT did not remove the VL but modified it, and eventually induced additional changes in the ultrastructure of the cell surface, such as the microvilli.

### 3.2. Effect of DTT Pretreatment of the Eggs on the Fertilization Process

**Figure 2:**
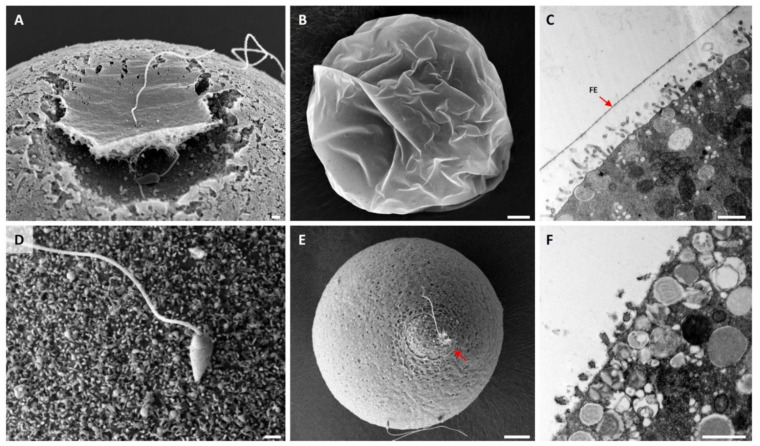
Ultrastructure of the egg surface immediately after fertilization. *P. lividus* eggs were fertilized and fixed for SEM (**A**,**B**,**D**,**E**) and TEM (**C**,**F**) analyses. (**A**–**C**) Control eggs fertilized in NSW. (**D**–**F**) Eggs preincubated (10 min) and fertilized in seawater containing 10 mM DTT. Images in (**A**,**D**) were captured by fixing the eggs 25 s after insemination. The images in all the other panels show the ultrastructure of the egg surface 1 min after insemination. Note that, in panel (**E**), most parts of a fertilizing sperm were not internalized yet, but were surrounded by the egg protrusion through the ruptured fertilization envelope (red arrow). Scale bars: panels (**B**,**E**), 10 μm; all other panels, 1 μm. FE, fertilization envelope.

Given that the DTT pretreatment partially disrupts the VL and modifies the microvilli, we examined how the same treatment would influence the fertilization process. To this end, *P. lividus* eggs preincubated in NSW for 10 min in the presence or absence (control) of 10 mM DTT were fertilized and fixed at certain intervals and were subjected to SEM and TEM analyses (Figure 2). By 25 s after insemination, it was already evident that the VL of the control egg had started to be elevated around the area where the fertilizing sperm was fused with the oolemma (Figure 2A). This change, induced by partially elevated nascent FE, created a dimple-like surface ([41,42], see Supplemental Video S1).

The detachment of the VL from the plasma membrane and the subsequent elevation of the VL to form the FE is a wave-like process resulting from the coordinated exocytosis of the cortical granules from the underlying egg cortex. By 1 min, the entire surface of the control egg was covered with FE (Figure 2B). On the other hand, fertilization in the eggs pretreated with 10 mM DTT appeared to proceed with some notable anomaly. By 25 s, the sperm was attached to the microvilli of the egg, but the initial elevation of the FE did not take place (Figure 2D). Although a more prominent fertilization cone was formed in the eggs fertilized in the presence of DTT, the eggs were covered with the thin and patchy looking vitelline layer (VL) (Figure 2E, also see the enlarged image in Figure S1). In agreement with this finding, the TEM image of the control egg obtained 1 min after fertilization exhibited a thick layer of FE (Figure 2C, arrow), which was absent in the eggs fertilized 10 min after the preincubation in 10 mM DTT (Figure 2F, see Supplemental Video S2). Judging from the discontinuous appearance of the VL (Figure 1F), the failure of VL to elevate and form FE in the DTT-pretreated eggs is thought to be related to loss of the integrity of the VL due to the breakage of the disulfide bonds in and among the molecules in the VL. In the latter case, the extruded contents of the cortical granules failed to accumulate in the perivitelline space as they were eventually lost to the media through the loose holes on the VL [7].

### 3.3. Effect of DTT Pretreatment of the Eggs on the Later Stage of Fertilization

**Figure 3:**
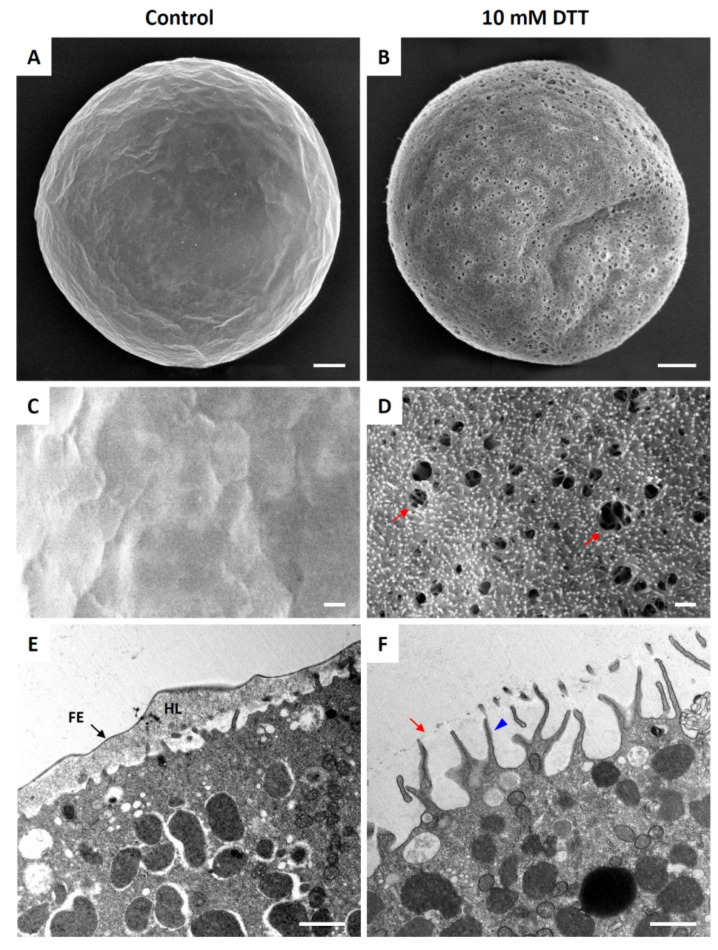
Ultrastructure of the egg surface at a later stage of fertilization. *P. lividus* eggs were fertilized and fixed 5 min later for SEM and TEM analyses. (**A**,**C**,**E**) Control eggs fertilized in NSW. (**B**,**D**,**F**) Eggs preincubated (10 min) and fertilized in seawater containing 10 mM DTT. Panels (**A**−**D**) show SEM images, while panels (**E**,**F**) represent results of TEM. Red arrows in panels (**D**,**F**) indicate the ruptured fertilization envelope. A blue arrowhead in panel F indicates the over-elongated microvilli. Scale bars: panels (**A**,**B**), 10 μm; all other panels, 1 μm. FE, fertilization envelope (arrows); HL, hyaline layer.

A fertilized sea urchin egg undergoes drastic reorganization at the cortex as the contents of the cortical granules are being extruded to the perivitelline space by exocytosis. Microvilli and the subplasmalemmal actin network are also rapidly reorganized at this time [26,43,44]. To test whether and how DTT affects the pattern of cortical rearrangement after fertilization, we preincubated *P. lividus* eggs in the presence or absence of 10 mM DTT prior to fertilization, and the resulting zygotes were fixed for SEM and TEM analyses 5 min after insemination (Figure 3). The whole-cell view of the SEM image showed that the entire surface of the egg fertilized in NSW was covered with a thick layer of FE (Figure 3A,C). By contrast, the eggs preincubated and fertilized in 10 mM DTT seawater did not form the normal FE (Figure 3B). Instead, numerous holes were observed on the surface, and the VL was barely elevated, so that the underlying microvilli were clearly visible within the holes (Figure 3D, arrows) and through the thin veiling VL. By 5 min, exocytosis of cortical granules and their extruded contents were heavily deposited to form the hyaline layer underneath the FE (Figure 3E). The exocytosis of the cortical granules was thought to be exhaustive by this time, as judged by their absence in the subplasmalemmal region. Indeed, the depth of the newly formed perivitelline space seen in the light microscopy was so immense that it often collapsed during the procedure of fixation, which was applied 5 min after insemination (Figure 3E). Similarly, most cortical granules also underwent exocytosis in the eggs pretreated and fertilized in the presence of 10 mM DTT, but the VL did not swell. There was only a faint remnant of elevated VL (Figure 3F, arrow). Notably, however, these eggs exhibited extensive elongation of the microvilli that stretched out to the VL (Figure 3F).

### 3.4. DTT Pretreatment of the Eggs Alters the Ca^2+^ Response at Fertilization

**Figure 4:**
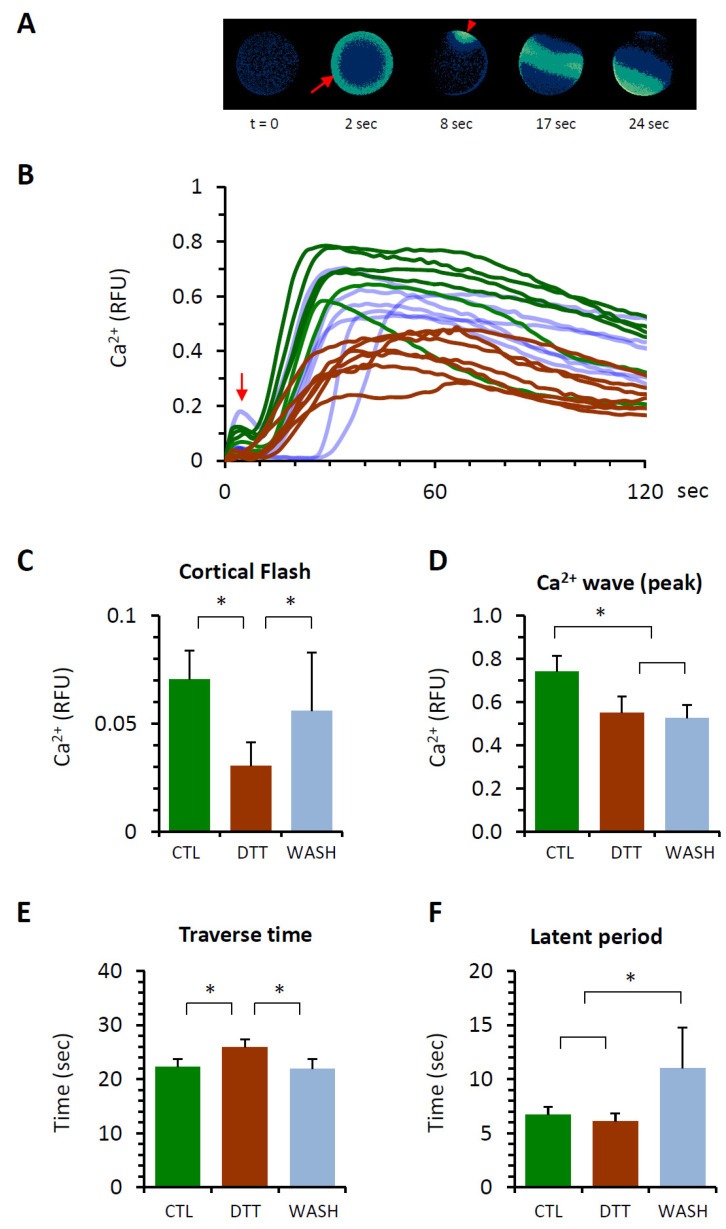
DTT pretreatment of the eggs alters the Ca^2+^ response at fertilization. *P. lividus* eggs were microinjected with calcium dye and preincubated in seawater with 10 mM DTT (10 min) prior to fertilization. As a control (CTL), eggs from the same batch were prepared in parallel and fertilized in NSW. For comparison, some eggs preincubated in the presence of 10 mM DTT were restored in normal seawater and fertilized (WASH). (**A**) Instantaneous increment of Ca^2+^ signals inside a control egg visualized in pseudocolour. The time point immediately before the first detectable Ca^2+^ signal was set as t = 0. The arrow indicates cortical flash (CF), and the arrowhead pinpoints the sperm interaction site on the egg where the Ca^2+^ wave is initiated. (**B**) Changes of the Ca^2+^ signals: green curves (CTL), brown curves (eggs fertilized in 10 mM DTT), and curves in pale blue (eggs fertilized after restoration in NSW, 10 min). The vertical arrow indicates the CF. Out of four independent experiments conducted, the result of one representative experiment was plotted for Ca^2+^ trajectories. Histograms in panels C−F represent comparisons of the egg groups with respect to the CF amplitude (**C**), the peak amplitude of the Ca^2+^ wave (**D**), the duration of the Ca^2+^ wave traversing the egg from the sperm interaction site to the antipode (**E**), and the time interval between the CF and the initiation of the Ca^2+^ wave (**F**). * Tukey’s post hoc test, *p* < 0.01. RFU, relative fluorescence unit.

The physiological consequence of the DTT pretreatment was assessed by examining the eggs’ Ca^2+^ response at fertilization. In the normal condition, fertilized sea urchin eggs displayed two modes of intracellular Ca^2+^ response (Figure 4A,B): (i) the synchronized Ca^2+^ increase near the plasma membrane of the entire egg surface (cortical flash (CF), and (ii) the Ca^2+^ wave that locally originates at the sperm-interaction site and propagates to the antipode. The rapid CF taking place immediately after the fertilizing sperm makes meaningful contact with the egg due to the Ca^2+^ influx into the egg through the voltage-gated calcium channels that can be modulated by the surrounding subcellular structures such as the microvilli [45,46,47]. On the other hand, the Ca^2+^ wave depends on the synthesis of Ca^2+^ mobilizing second messengers [20,22,24] and on the structural modification of cortical granules and vesicles [47,48].

The three groups of eggs (control, DTT, and WASH) responded to insemination with Ca^2+^ signals without showing any significant difference in latency: around 40 s after insemination in the given conditions. This observation suggests that the sperm receptivity in this regard was not changed much by the treatment with DTT. However, as expected from the modification of the egg surface, the amplitude of CF in the eggs preincubated and fertilized in 10 mM DTT seawater was significantly lower than that of the control eggs (Figure 4C,D). Similarly, the average peak amplitude of the Ca^2+^ wave in the eggs pretreated with DTT was significantly lower (0.55 ± 0.15 RFU, *n* = 24) in comparison with the control eggs (0.74 ± 0.15 RFU, *n* = 23, *p* < 0.01). In addition, the Ca^2+^ waves propagated more slowly in the eggs pretreated with 10 mM DTT, as judged by the time spans from the first appearance of the localized Ca^2+^ signal at the sperm interaction site to the moment of wave’s arrival at the antipode (Figure 4E, traverse time). While the average traverse time in the control eggs was 22.3 ± 3.0 s (*n* = 23), the corresponding value in the eggs pretreated with 10 mM DTT was 26.0 ± 2.8 s (*n* = 24, *p* < 0.01). Curiously, when the DTT-pretreated eggs were fertilized after being rinsed and restored in normal seawater, some of the Ca^2+^ signaling patterns altered by DTT were significantly reversed to assimilate those in the control eggs. Such a trend was found in the CF (Figure 4C), whose average amplitude in the eggs restored in NSW (WASH, 0.06 ± 0.05 RFU, *n* = 15) was comparable to the control (0.07 ± 0.03 RFU, *n* = 23), but significantly higher than the values in the eggs pretreated and fertilized in 10 mM DTT (0.23 ± 0.02 RFU, *n* = 19, *p* < 0.01). A similar reversal of the DTT-induced alteration in Ca^2+^ signaling pattern was also found in terms of the traverse time, which was shortened again (Figure 4E, WASH, 21.9 ± 3.7 s, *n* = 21, *p* < 0.01), but not in respect to the peak amplitude of the Ca^2+^ wave (Figure 4D, WASH, 0.53 ± 0.12 RFU, *n* = 21).

Another noteworthy observation is about the time interval between the CF and the first detectable Ca^2+^ signal at the site of sperm interaction, which is referred to as the “latent period” in this work (Figure 4F). This is the period when the fertilized egg already transmitting the intracellular signals such as membrane depolarization and Ca^2+^ influx prepares itself for the generation of the Ca^2+^ wave [24,31,47,49]. Despite the notable structural changes on the egg surface, the latent period of the Ca^2+^ response in the eggs preincubated and fertilized in seawater containing 10 mM DTT (6.1 ± 1.4 s, *n* = 19) was not much different from that of the control eggs (6.7 ± 1.5 s, *n* = 23). When the DTT-preincubated eggs were rinsed and restored in NSW before fertilization, however, the latent period for the Ca^2+^ response in these eggs was nearly doubled (11.3 ± 7.6 s, *n* = 15, *p* < 0.01) in comparison with both CTL and DTT. These results suggest that the alteration of the Ca^2+^-release systems induced by DTT pretreatment is reversed to some extent by the restoration of the eggs in NSW, but the recovery path may not be the same as the path of modification induced by DTT. In other words, the eggs in the group WASH are in another physical state and show a distinct response to the fertilizing sperm.

### 3.5. Effect of DTT Pretreatment of the Eggs on Monospermic Fertilization

As the DTT pretreatment significantly altered the topography of the egg surface, it was conceivable that such structural changes may induce polyspermy at fertilization. Indeed, the porous and patchy appearance of the egg’s VL at fertilization and its failure to form a strong FE all raised the possibility of supernumerary sperm entry. However, the results pooled from five different batches of *P. lividus* suggested that this is not the case. When the eggs were preincubated and fertilized in seawater containing 10 mM DTT, most eggs were still monospermic. The frequency of polyspermic fertilization was not significantly increased in comparison with the control eggs fertilized in NSW (Table 1). Furthermore, the number of egg-incorporated sperm had only a marginal increase in comparison with the control eggs, which was not statistically significant (Table 2). However, when the DTT-pretreated eggs were restored and then fertilized, the frequency of polyspermy went up to 60% (Table 1), and the number of egg-incorporated sperm at fertilization was greatly increased (2.38 sperm per egg, Table 2). This result was not the effect of prolonged incubation or agitation of the eggs, because in another control experiment, the eggs continuously exposed to 10 mM DTT, following the same protocol (i.e., 10 min incubation, media change, and another incubation for 10 min) exhibited mostly monospermic fertilization. The polyspermy rate in these eggs was merely 5%, which was virtually the same as that of the eggs fertilized in NSW.

The observation that polyspermy is induced in the DTT-pretreated eggs after the restoration in NSW may also be related to the fact that DTT can traverse the cell membrane and spread inside the cell. Indeed, a similar experiment with another reducing agent, TCEP, which does not penetrate the cell membrane [50,51,52], did not induce polyspermy in its presence. The eggs restored and fertilized in normal seawater after being preincubated with TCEP were also all monospermic at fertilization (Table 2). Hence, the cause of the polyspermy in these eggs might be attributable to some internal factors. One explanation could be the possibility that an abrupt shift of the pH of the external media (from pH 7.57 of DTT seawater to pH 8.1 of NSW) and the accompanying changes in microvillar morphology (elongation) due to the cytoplasmic alkalinization might have contributed to polyspermy [53], in a sense that elongated microvilli could increase the capacity of the egg to fuse with sperm [54].

### 3.6. F-Actin Mobilization during Egg Activation Is Inhibited by DTT

**Figure 5:**
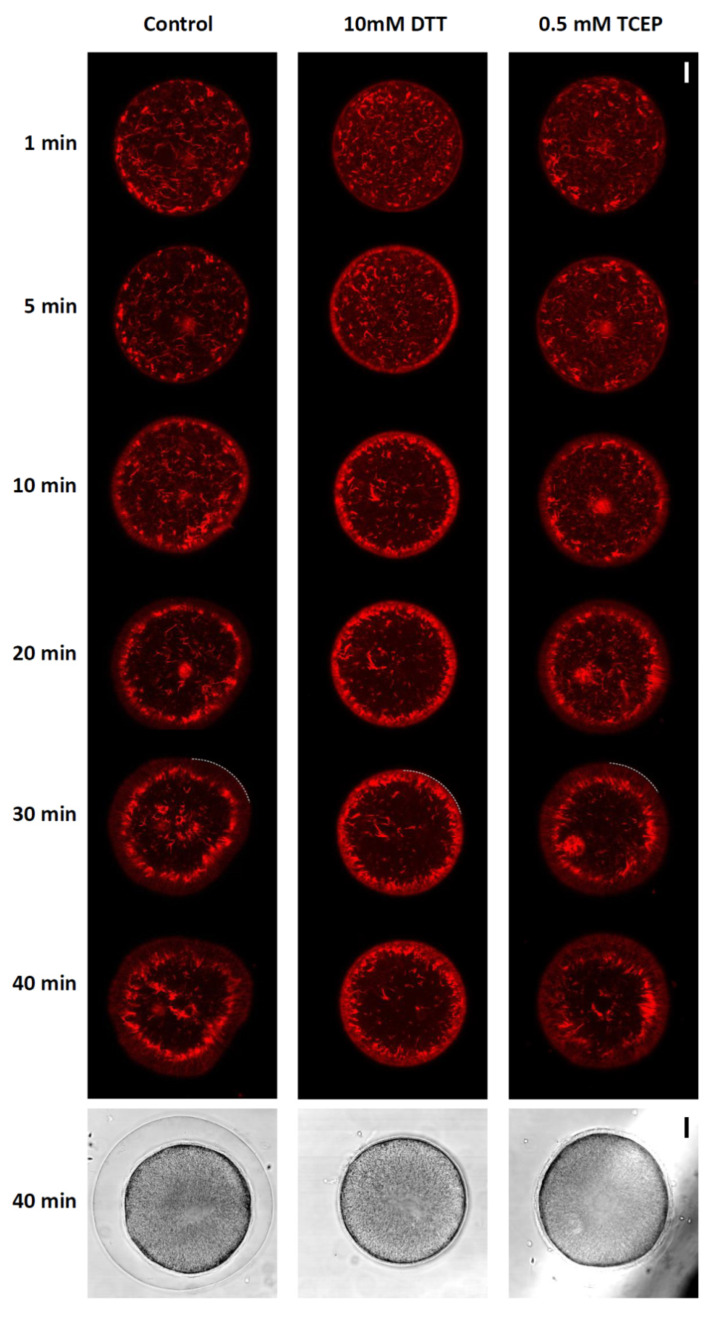
F-actin mobilization during egg activation is inhibited by DTT, the membrane-permeant reducing agent. *P. lividus* eggs were microinjected with F-actin dye, AlexaFluor 568-phalloidin, and preincubated in seawater in the presence or absence (control) of 10 mM DTT for 10 min prior to fertilization. For comparison, some of the control eggs were exposed to another type of reducing agent that does not penetrate cell membrane, tris(2-carboxyethyl)phosphine (TCEP). The changes of the actin cytoskeleton following fertilization were monitored by confocal microscopy at intervals. The moment of insemination was set as t = 0. The white dotted curves drawn on the images at 30 min delineate the borders of the egg surface. To visualize the fertilization envelope, the same AlexaFluor 568-phalloidin eggs were shown in the bright field view 40 min after insemination. These images are representative of at least two independent experiments. Scale bar, 10 μm.

In a series of previous studies on starfish and sea urchin eggs, we demonstrated that polyspermy is closely linked to an anomaly in the actin cytoskeleton of the egg cortex. Indeed, when the actin cytoskeleton was artificially altered through the use of actin-targeting toxins and chemicals, an actin-binding protein cofilin, anti-depactin antibody, nicotine, or even by “ageing”, the eggs showed a strong tendency of polyspermic fertilization [27,29,39,55,56,57,58]. At variance with these experimental conditions that caused cytoskeletal alterations, the pretreatment of *P. lividus* eggs with DTT or TCEP in our experimental condition did not induce discernable changes in the structure of the cortical actin cytoskeleton (data not shown). This may be one of the reasons why the DTT- and TCEP-pretreated eggs were not prominently polyspermic (Table 1 and Table 2). Nonetheless, it is possible that the concerted rearrangement of the actin cytoskeleton taking place in the fertilized eggs during egg activation [25,28,29] may not be influenced by the DTT pretreatment. In other words, the reducing agent may not have a profound effect on the actin filaments inside a quiescent egg, but it may still affect the mobilization kinetics in post-fertilization eggs. We tested this idea by microinjecting the eggs with a fluorescent F-actin probe (AlexaFluor 568-Phalloidin) before the DTT and TCEP pretreatment and fertilization (Figure 5).

In the control eggs fertilized in NSW, the subplasmalemmal F-actin started to take an orderly form and left the area to move toward the inner cytoplasm (*n* = 15). This was more evident when the locations of the thick ring-shaped F-actin clusters were compared with the border of the egg surface in the egg images 30 min after fertilization. In the control egg, by then, there was at least 10–15 μm distance between the F-actin clusters and the boundary of the egg surface (marked by dotted curve, Figure 5). Hence, the actin filaments underneath the plasma membrane were evacuated from the subplasmalemmal zone. However, this characteristically concerted centripetal migration of the cortical actin filaments was mostly blocked in the eggs preincubated and fertilized in 10 mM DTT (*n* = 12), as the ring-shaped F-actin clusters had a strong tendency to remain in the vicinity of the plasma membrane from 5 min and on. At 30 min, they were still very close to the plasma membrane (Figure 5, see the dotted curve for the DTT-pretreated eggs). This inhibitory effect of DTT on the inward movement of the cortical actin filaments may be attributed to its reducing power inside the egg’s cytoplasm, but not on the external surface of the egg. A similar reducing agent unable to permeate the cell membrane, TCEP, did not have such an effect. In these eggs (*n* = 8), the ring-shaped F-actin clusters were relocated toward the inner cytoplasm just like in the control eggs (Figure 5, dotted curve). The lack of the inhibitory effect on F-actin mobilization displayed by TCEP is not a matter of the low efficacy of the agent. When added to the media, 0.5 mM TCEP blocked the formation of the FE as effectively as the 10 mM DTT did (Figure 5, see the egg images in the light transmission view). These results suggest that the orderly migration of the egg’s cortical actin filaments following fertilization is sensitive to the redox status of the cytoplasm.

### 3.7. Effect of DTT Pretreatment on the Late Events of Fertilization

**Figure 6:**
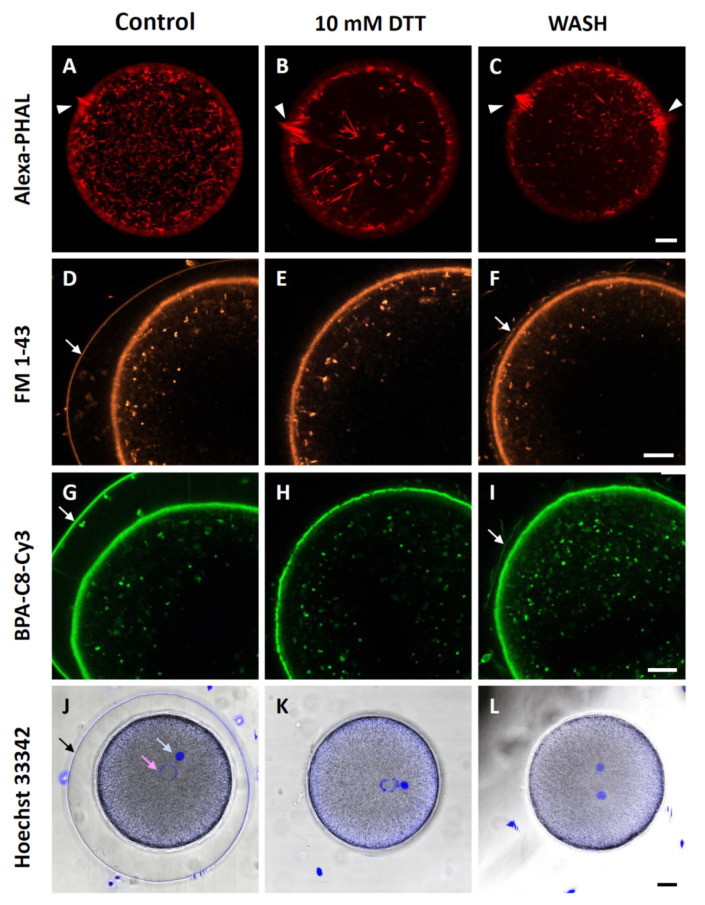
Effect of DTT pretreatment on the late events of fertilization. (**A**–**C**) *P. lividus* eggs were microinjected with F-actin dye, AlexaFluor 568-phalloidin, and preincubated in seawater in the presence or absence (control) of 10 mM DTT for 10 min prior to fertilization. After 10 min, the actin cytoskeleton of the fertilized eggs was visualized by confocal microscopy. The fertilization cones are marked by arrowheads. (**D**–**I**) Eggs pretreated in the same conditions as above (but without F-actin dyes) were fertilized and incubated in the presence of FM 1-43 (**D**–**F**) or BPA-C8-Cy3 (**G**–**I**). White arrows indicate the fertilization envelope and its remnants. (**J**–**L**) Hoechst 33,342 introduced with sperm visualized the male and female pronuclei of the zygotes in the given conditions, as indicated by pale blue and pink arrows, respectively. The black arrow indicates the fertilization envelope. These images are representative of at least two independent experiments. Scale bar, 10 μm.

In the eggs preincubated and fertilized in 10 mM DTT seawater, the anomaly of the actin cytoskeleton was also observed in the fertilization cone, the F-actin-based cytoskeletal apparatus specializing in the capture and engulfing of the fertilizing sperm. On the confocal plane where the fertilization cone was located, the control eggs fertilized in NSW (*n* = 15) after microinjection of AlexaFlour 568-Phalloidin displayed an array of thick actin fibers oriented perpendicular to the plasma membrane (Figure 6A). In the eggs preincubated and fertilized in the presence of 10 mM DTT (*n* = 12), however, the morphological symmetry of the fertilization cone was lost, and the aggregation of the thick actin fibers was visibly exaggerated amid other thick actin fibers in the inner cytoplasm, which were similar to the stress fibers (Figure 6B, arrowhead). On the other hand, in the eggs preincubated with DTT but washed (*n* = 12) and restored in NSW prior to fertilization, there were often multiple fertilization cones (Figure 6C).

Although the vitelline layer was partially disrupted and certain aspects of the dynamic property of the egg’s cortical actin cytoskeleton were altered by DTT (e.g., centripetal migration of actin filaments), the endocytotic activity at the plasma membrane that retrieves the membranes following cortical granule exocytosis [59,60] appeared to be normal. When the eggs were stained in the media by adding the fluorescent membrane probe FM 1–43 that sticks to the outer leaflet of the plasma membrane [61,62], numerous internalized vesicles appeared in the inner cytoplasm of the eggs 10 min after fertilization (Figure 6D, *n* = 7). Evidently, the eggs preincubated and fertilized in the presence of 10 mM DTT (*n* = 7) also displayed virtually the same results (Figure 6E), and DTT washing and restoration in NSW (*n* = 7) did not make much difference to the membrane retrieval after fertilization. Nonetheless, it is noteworthy that, in these DTT-preincubated eggs restored in NSW before fertilization, there was some modest formation of FE (Figure 6F, arrow). This result was also corroborated by another fluorescent probe that is derivative of synthetic polyamine [63], which is thought to have a tendency to bind to negatively charged macromolecular surfaces such as the plasma membrane and actin filament meshwork [64,65]. Applied in the media around echinoderm eggs, this fluorescent probe appears to stain predominantly the jelly coat of starfish oocytes (Santella et al. unpublished data). For *P. lividus* eggs at fertilization, this novel probe BPA-C8-Cy3 exhibited intense fluorescent labelling of the VL, FE, and subplasmalemmal regions, as well as the membraneous structures within the cytoplasm and the perivitelline space (Figure 6G, *n* = 10). In the DTT-preincubated eggs that were restored in NSW before fertilization (*n* = 10), modest but evident formation of the FE was detected, as with FM 1-43 (Figure 6I, arrow). Hence, these results suggest that certain aspects of cellular changes induced by DTT can be reversed to some extent, but not completely.

Importantly, we noted that BPA-C8-Cy3 produced evidently thinner and occasionally interrupted labelling at the junction of the plasma membrane and the VL in the eggs pretreated and fertilized in the seawater containing 10 mM DTT (*n* = 10, compare Figure 6H with Figure 6G,I). Again, after washing and restoration in NSW, the fluorescent labelling at the VL and plasma membrane came back to the appearance of the control eggs (Figure 6I). By contrast, such reversed variations in the level of fluorescence were not observed in the eggs labelled with the membrane-specific probe FM 1-43 (Figure 6D–F). Subtle as it might look, this was the striking difference between FM 1-43 and BPA-C8-Cy3. These observations in comparison with FM 1-43 are compatible with the idea that BPA-C8-Cy3 selectively visualizes the VL, which was occasionally interrupted in the eggs pretreated with DTT.

We then tested whether or not DTT pretreatment of the eggs affected the migration of male and female pronuclei. Following the experimental procedure described in Materials and Methods, the male and female pronuclei were fluorescently labelled in the zygote (Figure 6J). The densely labelled male pronucleus appeared more compact than the female pronucleus, and the distance between the two pronuclei was not much different in the zygotes obtained from DTT-preincubated eggs either with or without restoration in NSW (Figure 6K,L). Thus, taken together with the results in Figure 5, these observations suggest that exposure of the eggs to DTT in the condition that blocks the centripetal movement of the subplammalemmal actin filaments actually has little effect on pronuclei movement, the process that has been reported to be mainly dependent on the transport system, largely based on microtubules [66,67].

### 3.8. Effect of DTT Pretreatment of the Eggs on Early Embryonic Development

**Figure 7:**
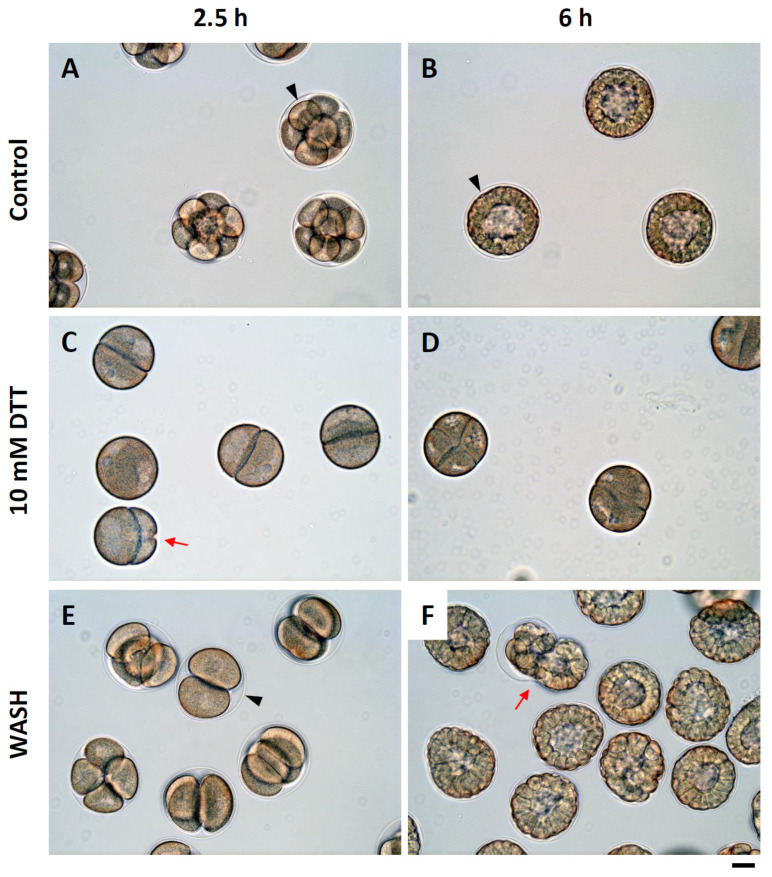
DTT pretreatment of the eggs induces anomalies during early development. (**A**–**D**) *P. lividus* eggs were incubated in the presence or absence (control) of 10 mM DTT for 10 min and fertilized. (**E**–**F**) Some of the DTT-pretreated eggs were rinsed and restored in NSW (10 min) and fertilized (WASH). The cleavage and development of the embryos in each condition were monitored by light microscopy. Arrowheads indicate the FE. Note that the FE covering the control embryo is still intact at 6 h (**B**), where it is totally absent in the embryos fertilized in the presence of DTT. The FE is evident again in the embryos derived from the eggs restored in NSW prior to fertilization (**F**). Red arrows indicate signs of leakage in the embryo from the ruptured FE. Scale bar, 10 μm. FE, fertilization envelope. The number of fertilized eggs examined for each set of experiments was 100. Representative results from one of the three independent experiments are shown.

The eggs restored in NSW after being preincubated in DTT exhibited a certain degree of plasticity in terms of Ca^2+^ signaling, F-actin mobilization, and FE formation during fertilization, albeit a high frequency of polyspermy. To explore this issue a step further, we examined the early phase development of those embryos derived from the monospermic zygotes obtained from each experimental condition. By 2 h and 30 min, the control eggs fertilized in NSW were well into the 8-cell stage. The cleavage also appeared quite synchronous (Figure 7A). In contrast, when the eggs were preincubated and fertilized in the presence of 10 mM DTT, the majority of embryos were at the two-cell stage, suggesting that the cleavages of the zygote had been significantly delayed. Indeed, in some cases, it appeared that the individual daughter cells had difficulties completing cytokinesis, as judged by the partially formed cleavage furrow (Figure 7C, arrow). By 6 h post-fertilization, the control embryos were at the early blastula stage (Figure 7B), but the embryos deriving from the eggs fertilized in the presence of DTT were still at the four-cell stage with some cleavage anomaly (Figure 7D). This problem of cytokinesis seemed to be overcome when the DTT-preincubated eggs were restored and fertilized in NSW. However, 2.5 h after insemination, the embryos were mainly at the four-cell stage (Figure 7E), a clear sign of delay in comparison with the control embryos. Six hours after insemination, the embryos derived from the eggs restored and fertilized in NSW showed varying blastomere sizes, abnormal cleavage, and their leaking from the ruptured evanescent FE (Figure 7F, red arrow). 

## 4. Discussion

As a part of the extracellular matrix, the VL is thought to play important roles during fertilization and early embryonic development. In this study, we induced partial disruption of the VL in *P. lividus* eggs through the use of reducing agents that cleave intra- and inter-molecular disulfide bonds. The experimental condition (10 mM DTT, 10 min) of the egg pretreatment was mild enough not to cause total removal of the VL, as judged by the images in the TEM and SEM analyses, but introduced subtle ultrastructural changes on the egg surface. The VL was evidently interrupted (Figure 1) and failed to transform into FE at fertilization (Figure 2). This is presumably because the VL was not physically sealed up due to the patchy structure induced by DTT, and thereby the osmotic pressure failed to build up in the perivitelline space, while proteins and other materials extruded from the cortical granules were eventually lost to the external medium [7,41,68].

The lack of VL integrity is likely to have altered the mechanical property of the egg surface because the plasma membrane is intimately ensheathed by the VL. Furthermore, the structure of the microvilli underlying the VL and the plasma membrane was appreciably modified in the eggs pretreated with 10 mM DTT. Another indication of cytoskeletal changes in the outer cytoplasmic region of the eggs fertilized in seawater containing DTT was the lack of cytoplasmic contractility [69], which prevented the formation of the dimple-like structure where the sperm fused with the oolemma (Figure 2A, Supplemental Videos S1 and S2) [42,70]. Moreover, the appearance of an extraordinarily enhanced fertilization cone in these eggs (Figure S1), together with the delayed sperm incorporation (Figure 2E), underline the alteration of the F-actin dynamics necessary to engulf the sperm [71].

The plasticity of *P. lividus* eggs was also manifested when the DTT-pretreated eggs were restored in NSW. While the eggs fertilized in the presence of DTT exhibited some anomalies (e.g., repressed Ca^2+^ response and failed FE elevation), the eggs pretreated with DTT but restored in NSW experienced less alterations in sperm-induced Ca^2+^ signals in terms of CF and traverse time. Likewise, the reversal of the altered Ca^2+^ response at fertilization was also observed in *P. lividus* eggs that were restored in NSW after being exposed to low salinity and other conditions [72,73], as well as in *Arbacia lixula* eggs that were incubated in hypertonic salinity and then inseminated in NSW [74]. In the eggs restored in NSW after DTT pretreatment, however, the physicochemical and structural changes of the cortex were accompanied by polyspermy at fertilization, as well as some anomaly in embryonic development (Figure 7). The centripetal translocation of the subplasmalemmal actin filaments during egg activation has been observed in both sea urchin and starfish [25,28,29,58]. It is conceivable that this orderly translocation of the actin filaments may contribute to cytoplasmic sorting and reorganization of the egg organelles in preparation for the subsequent cleavage. The eggs fertilized in the presence of DTT failed to exhibit the centripetal translocation of F-actin, and these eggs had significant delay and problems in the cleavages. Beyond this, the precise role and physiological significance of the translocation of F-actin in activated eggs are still largely unknown. Nevertheless, the results of our study using the eggs microinjected with AlexaFluor568-phalloidin and exposed to the two different reducing agents suggested that this translocation of cortical actin filaments may be sensitive to the cytoplasmic redox state of the eggs, as judged by its inhibition by DTT, the membrane-permeant reducing agent. In support of this idea, TCEP, which is unable to penetrate the cell membrane, had no such inhibitory effect on the reshuffling of cortical actin filaments (Figure 5). Nevertheless, further investigation is needed to understand to what extent the redox state of the egg cytoplasm is affected by the given conditions of DTT treatment and to identify the major targets of DTT that are accountable for our observations. Indeed, the importance of exquisite control of the redox state in oocytes and embryonic cells is increasingly being appreciated [75]. On the other hand, our observation of the apparently normal migration of female and male pronuclei in the eggs pretreated and fertilized in seawater containing DTT (Figure 5C) suggests that it is not likely that the centripetal movement of the cortical actin filaments makes a significant contribution to the transportation of the egg-engulfed sperm. While the molecular mechanism through which DTT inhibits the translocation of the cortical actin filaments is not known, it has been shown that myosin can form disulfide bonds within its regulatory light chain [76]. Thus, it is tempting to speculate that a DTT-induced shift of the myosin pool to its reduced form might have interfered with the translocation of the cortical actin filaments. In support of this idea, the inner cytoplasm of the eggs fertilized in the presence of DTT showed a conspicuous occurrence of stress fibers during egg activation, which might be a sign of deregulated actin-myosin interaction (Figure 6B). Alternatively, it is noteworthy that actin itself can exist as a dimer linked by disulfide bonds in vitro [77,78,79]. If such a transitory dimerization of actin molecules utilizing cysteine residues takes place inside the eggs and contributes to the centripetal translocation of microfilaments, it cannot be ruled out that actin itself might be the direct target of DTT.

The findings in this study raise a few more significant points on the cell biology of oocytes and eggs. In conventional practice, to follow the cell surface events at fertilization, eggs were often attached to polylysine-coated plates [80] or deprived of their jelly coat and VL [15]. These so-called “denuded eggs” may better adhere to the solid substrate such as slide glass, and were relatively immobilized during egg activation because of the lack of the FE elevation. This feature was advantageous for morphological analysis and other experiments. As the veil of VL was removed, it was relatively easier to disclose the sperm’s interaction with the microvilli in SEM. The ultrastructural images obtained with such methods were strikingly similar to the ones that we obtained with the DTT pretreatment [81]. However, it should be underscored that this is not a natural view and may be quite deviant from reality [82]. Instead, the control eggs visualized with SEM in our study were in their natural state, freely suspended in NSW until they were fixed for morphological analyses. As the DTT-pretreated eggs varied from the ones in natural conditions, as shown here in the analyses of their cell physiology, it bears emphasis that the experimental results obtained from those denuded or modified eggs may deviate from what really happens in natural conditions. This small but important methodological difference should be recognized. Secondly, we note that the significant alteration in the structure of the VL and the defective formation of the FE did not lead to polyspermy in the eggs pretreated with DTT. Combined with the previous findings [29,33,39], this observation again suggests that the FE may not be the decisive factor in preventing polyspermy in echinoderm eggs. Our result is also compatible with the idea that, if preferential binding and penetration sites exist on the VL, such receptors are not sensitive to DTT treatment by allowing the incorporation of the first sperm arriving at the egg surface. Finally, with the evidence of selective labelling at the VL and plasma membrane junction, we suggest that BPA-C8-Cy3 could be used as a convenient fluorescent probe in visualizing the VL of the egg.

## 5. Conclusions

Quiescent it may seem, a sea urchin egg is at the tight equilibrium for maintaining the homeostasis of intracellular Ca^2+^, pH, and redox state. Here, when the reducing agent DTT was added to the incubation media, the sea urchin egg displayed altered Ca^2+^ responses and F-actin translocation at fertilization. While the eggs fertilized in this condition were mostly monospermic, their development was severely impaired if DTT was still present in the media. When DTT was removed prior to fertilization, the eggs were overly polyspermic. All of these changes took place while the alteration of the VL was rather moderate; that is, the VL was not completely removed. In view of the fact that TCEP, another reducing agent but membrane-impermeant, did not interfere with the translocation of actin filaments following fertilization, the intracellular redox state is thought to be an important parameter that contributes to egg activation. Compared with the well characterized intracellular Ca^2+^ signaling in fertilized eggs, the roles played by intracellular pH and the redox state have been far less explored, but deserve more intense studies in the future.

## Figures and Tables

**Table 1:** Frequency of polyspermy (%) in *P. lividus* pretreated with various conditions.

Frequency (%)	NSW	10mM DTT	DTT/WASH	0.5mM TCEP	TCEP/WASH
**Mean**	4	11	60 *^,^^#^	0	0
**SD**	4.18	21.9	35.9	0	0
**N**	5	5	5	1	1

Note: Each trial in a given condition comprises 20 eggs. * *p* < 0.01 in Tukey test in comparison with NSW. # *p* < 0.05, with DTT.

**Table 2:** Number of egg-incorporated sperm inside the *P. lividus* eggs fertilized in various treatments.

Sperm Per Egg	NSW	10mM DTT	DTT/WASH	0.5mM TCEP	TCEP/WASH
**Mean**	1.04	1.13	2.38 *	1	1
**SD**	0.2	0.42	1.62	0	0
**n**	100	100	100	20	20

Note: * *p* < 0.00001 in U-test in comparison with the control (fertilization in natural seawater, NSW).

## Data Availability

Not applicable.

## Supplementary Materials

The following are available online at https://www.mdpi.com/article/10.3390/cells10123573/s1, Figure S1: Enlarged view of DTT-pre-treated sea urchin egg 1 min after insemination, Video S1: Fertilization of sea urchin control eggs, Video S2: Fertilization of DTT pretreated sea urchin eggs, File S1: Synthesis of the fluorescent molecular probe BPA-C8-Cy3.
