# Supplementary material for: Effects of Dithiothreitol on Fertilization and Early Development in Sea Urchin
Source: Cells. 2021 Dec 17;10(12):3573. doi: 10.3390/cells10123573 (PMC8700669; doi:10.3390/cells10123573)

Synthesis of the fluorescent molecular probe BPA-C8-Cy3.

We describe here an improved procedure for the synthesis of the fluorescent molecular labelling probe BPA-C8-Cy3 used in the present work and reported earlier in Nedeva I, Koripelly G, Caballero D, Chieze L, Guichard B, Romain B, Pencreach E, Lehn J-M, Carlier M-F, Riveline D. Synthetic polyamines promote rapid lamellipodial growth by regulating actin dynamics. Nat Commun 2013; 4:2165-11; PMID:23893126; http://dx.doi.org/10.1038/ncomms3165.
Reagents and solvents were purchased from Sigma-Aldrich, TCI, Fluorochem and were used without further purification. Deuterated solvents were purchased from Euriso-TOP or Sigma-Aldrich.
. **Flash column chromatography** was on a Biotage Isolera (column SNAP Ultra C18).
Nuclear Magnetic Resonance (NMR).
***^{1}H-NMR* spectra** NMR spectra were recorded on a Bruker Avance III HD 400 or Bruker Avance Neo 500 MHz NMR spectrometer. Chemical shifts are reported in parts per million (ppm) from low to high frequency using residual protonated solvent signals as reference (for ^{1}H NMR spectra $CDCl_3$=7.283 ppm, $C_2D_2Cl_4$=6.0 ppm; for ^{13}C NMR spectra $CDCl_3$=77.03 ppm). Coupling constants (*J*) are reported in hertz (Hz). The multiplicity of the 1H signals are indicated using the following standard abbreviations: s = singlet, d = doublet, t = triplet, dd = double doublet, q = quartet, m = multiplet, br = broad, ddd = doublet of double doublets. NMR signals are reported in terms of chemical shift (*δ*), multiplicity, coupling constants (*J*), relative integral, and assignment, in that order. Spectra were recorded at 25°C.
Mass Spectrometry. High resolution ESI mass spectra were obtained in-house at the Institute of Science and Supramolecular Engineering (ISIS) located in Strasbourg, on a Thermofisher Exactive Plus EMR Orbitrap mass spectrometer.

Synthesis of N,N,N',N'[tetrakis(aminopropyl) octamethylenediamine BPA-C8

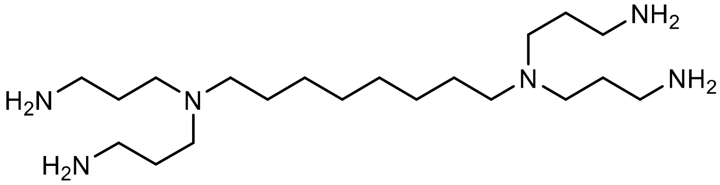

To a solution of 3,3',3'',3'''-(octane-1,8-diylbis(azanetriyl))tetrapropanenitrile (3.0 g, 8,45 mmol) in 240 ml of EtOH/THF (4/1) was added Raney-nickel (8,5 g, 144 mmol) as a 50% suspension in water. The solution was degassed twice under argon. Was added 50 ml NaOHaq (2M) and the reaction mixture was degassed again and stirred under 1 atm of H_2 at room temperature for 24 h. The mixture was filtered through a pad of Celite, washed with EtOH (70 ml) and the solvents were removed under reduced pressure. The residue was dissolved in a mixture of water and $CHCl_3$ (1/2) (150ml). The aqueous layer was extracted with $CHCl_3$. The organic layers were dried over Na_2SO_4, filtered and the solvent evaporated under reduced pressure. Compound was obtained as light-yellow oil (2,8 g). (90%)

HRMS (ESI+) calculated for [C20H50N6]: 187.2043 Da, found [$M^{+}2H^{+\cdot}$] 187.2040 *m/z*.

Synthesis of Cy3

Synthesis of **N-(3-Bromopropyl)triethylammonium bromide:**

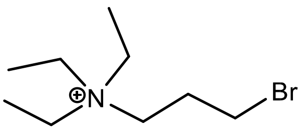

To a degassed solution of 1,3-Dibromopropane (30.0 g, 146 mmol, 2 equiv.) in 110 ml of toluene is added slowly triethylamine (7.52 g, 75 mmol) and heated at 100 ºC for 4 h After cooling the white precipitated obtained and washed with toluene and ether and dried under reduced pressure to obtain the bromopropyltriethylammonium salt (15,2 g, 69%) as a white solid;

^{1}H NMR (500 MHz, H_2O+D_2O) δ 3.44 (t, *J* = 6.0 Hz, 2H), 3.28 – 3.12 (m, 8H), 2.22 – 2.12 (m, 1H), 1.18 (ddd, *J* = 9.0, 5.3, 1.9 Hz, 9H).

^{13}C NMR (126 MHz, H_2O+D_2O) δ 52.74, 29.24, 24.12, 6.60.

HRMS (ESI+) calculated for [$C_9H_{21}N_2BrN$]: 222.0852 Da, found [$M^{+\cdot}$] 222.0846 *m/z*.

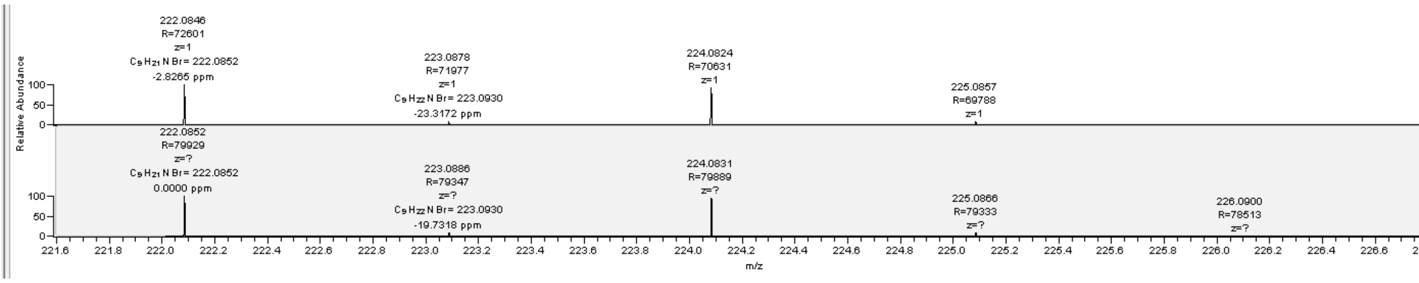

Synthesis of **1-((3-triethylammonium)propyl)-2,3,3-trimethylindolium diibromidetriethylammonium bromide:**

A mixture of 2,3,3-trimethylindolenine (2,9 g,18.21mmol) and N-(3-bromopropyl)triethylammonium bromide (5.5 g, 24.69 mmol) was heated at 150 ºC for 2h and cooled to room temperature . The deep red solid obtained was dissolved in CH_2Cl_2 and precipitated with Et_2O. The product was obtained as a dark red foamy solid (3,86 g, 70%)

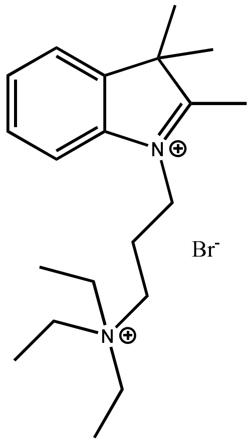

^1H NMR (500 MHz, H_2O+D_2O) δ 7.67 (d, *J* = 7.3 Hz, 2H), 7.57 (p, *J* = 7.4 Hz, 2H), 4.50 (t, *J* = 8.1 Hz, 1H), 3.40 – 3.33 (m, 2H), 3.23 (q, *J* = 7.3 Hz, 6H), 2.77 (d, *J* = 8.7 Hz, 2H), 2.30 – 2.24 (m, 2H), 1.49 (s, 6H), 1.19 (t, *J* = 7.3 Hz, 9H).

HRMS (ESI+) calculated for [$C_{20}H_{34}N_2$]: 151.1356 Da, found [$M^{2+\cdot}$] 151.1352 *m/z*.

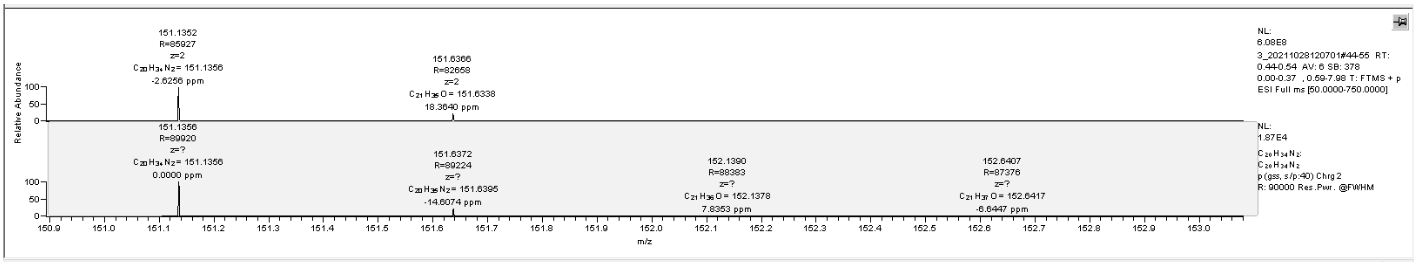

Synthesis of **1-(5-Carboxypentyl)-2,3,3-trimethylindolium bromide**

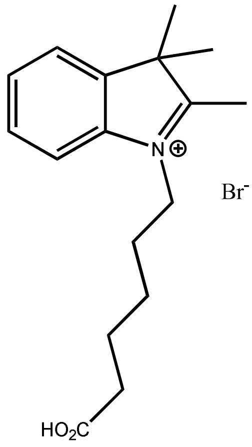

A mixture of 2,3,3-trimethylindolenine (3.0 g, 18.8 mmol, 1 equiv.) and 6-bromohexanoic acid (5.16 g, 26.4 mmol, 1.4 equiv.) in 1,2-dichlorobenzene (50 mL) was heated at 110 ºC for 12 h. The solution was cooled to room temperature and the solvent was removed under reduced pressure. The viscous oil obtained was diluted with Et_2O/CH_2Cl_2 (1/1) and the resulted precipitate was filtered and washed with ether to obtain carboxypentyl trimenthylindolium bromide (3.77 g, 73%) as a pink dark solid.

^{1}H NMR (500 MHz, H_2O+D_2O) δ 7.70 – 7.61 (m, 2H), 7.58 – 7.49 (m, 2H), 4.37 (t, *J* = 7.6 Hz, 2H), 2.71 (s, 2H), 2.25 (t, *J* = 7.3 Hz, 2H), 1.88 (p, *J* = 7.6 Hz, 2H), 1.55 (p, *J* = 7.4 Hz, 2H), 1.46 (s, 6H), 1.35 (q, *J* = 8.3 Hz, 3H).

HRMS (ESI+) calculated for [$C_{17}H_{24}O_2N$]: 274.1802 Da, found [$M^{+\cdot}$] 274.1792 m/*z*.

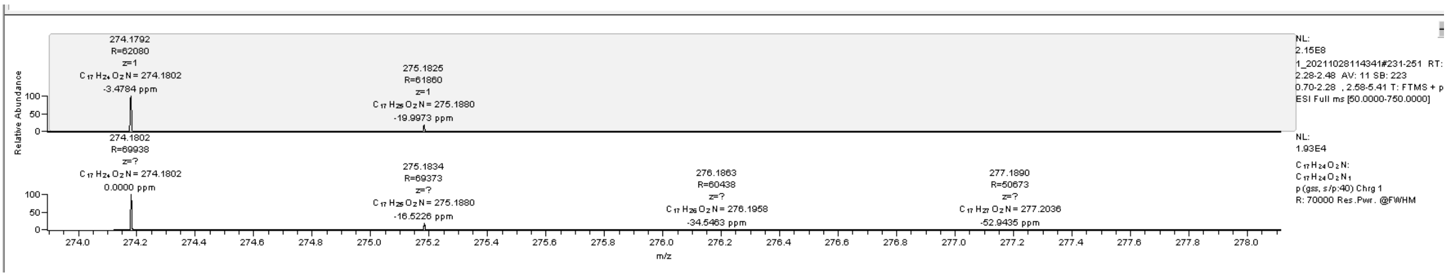

.

Synthesis of **1-(5-carboxypentyl)-3,3-dimethyl-2-[(E)-2-(phenylamino)ethenyl]-3H-indol-1-ium acetate**

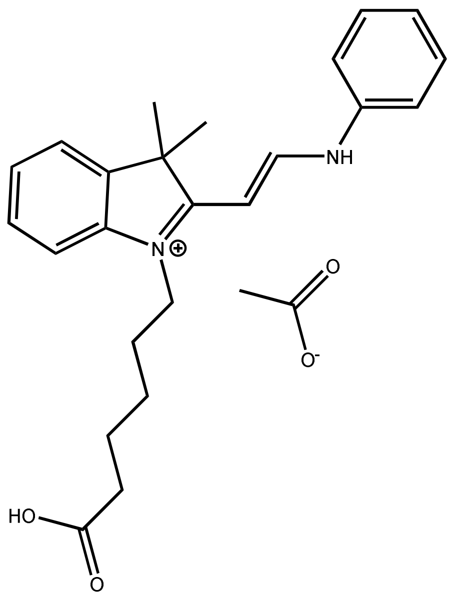

A mixture of 1-(5-Carboxypentyl)-2,3,3-trimethylindolium bromide (3.0 g, 10,9 mmol) and N,N´-diphenylformamidine (4.3 g, 21,9 mmol,) in 30 ml of acetic acid was heated at relux for 2.5 h. The resulted orange-red solution was cooled to room temperature and the solvent was evaporated under reduced pressure. The residue obtained was purified by flash chromatography, Biotage, column SNAP Ultra C18, MeOH/H_2O with 10 % of acetic acid to give the title compound (3,1 g, 73%) as an orange solid.

^{1}H NMR (500 MHz, H_2O+D_2O) δ 8.47 (d, *J* = 12.6 Hz, 1H), 7.48 (d, *J* = 7.6 Hz, 1H), 7.40 (q, *J* = 8.4 Hz, 3H), 7.27 (td, *J* = 15.4, 7.3 Hz, 4H), 6.04 (d, *J* = 12.6 Hz, 1H), 4.05 (t, *J* = 7.2 Hz, 2H), 2.21 (t, *J* = 7.3 Hz, 2H), 1.78 (p, *J* = 7.4 Hz, 2H), 1.61 (s, 6H), 1.53 (p, *J* = 7.5 Hz, 2H), 1.32 (p, *J* = 8.0 Hz, 2H).

HRMS (ESI+) calculated for [$C_{24}H_{29}O_2N_2$]: 377.2224 Da, found [$M^{+\cdot}$] 377.2211 *m/z*.

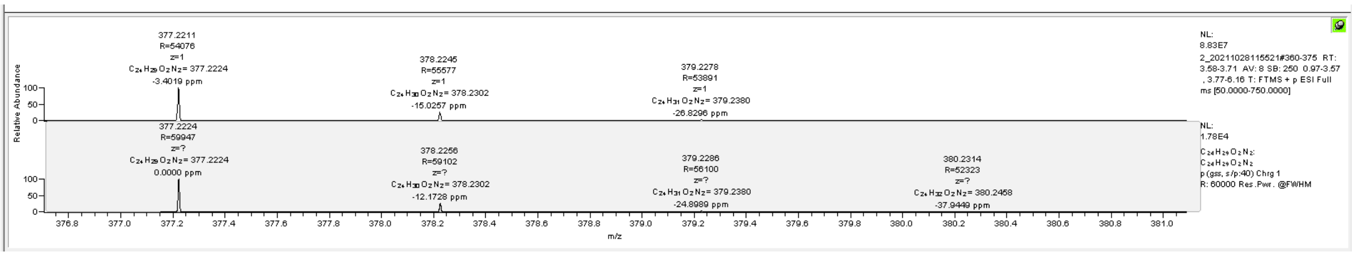

Synthesis of the **Cy3** dye fragment

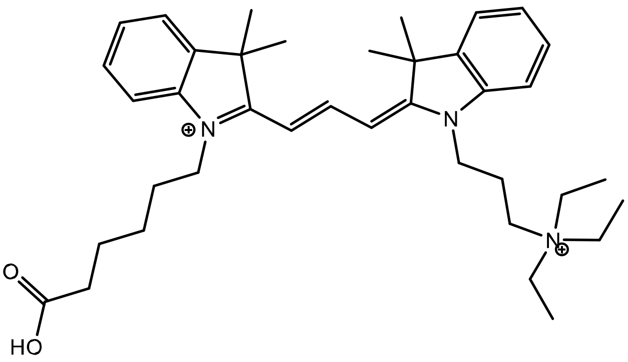

A solution of 1-(5-carboxypentyl)-3,3-dimethyl-2-[(E)-2-(phenylamino)ethenyl]-3H-indol-1-ium acetate (1 eq., 244 mg, 0.647 mmol) in pyridine (6.31 mL) was added acetic anhydride (12.4 eq., 818 mg, 0.753 mL, 8.02 mmol) and the yellow mixture was stirred for 5 mins at rt. 1-((3-triethylammonium)propyl)-2,3,3-trimethylindolium diibromidetriethylammonium bromide (1 eq., 195 mg, 0.647 mmol) was then added and the resulting pink mixture was stirred for 2h at the same temperature.

The solvent was then removed under reduced pressure and the residue was dried under vacuum. The crude product was purified by automatic flash chromatography Biotage Isolera, column SNAP Ultra C18, MeOH/H_2O with 10 % of acetic acid.

^{1}H NMR (500 MHz, H_2O+D_2O) δ 8.46 (s, 0H), 7.48 (t, *J* = 7.4 Hz, 2H), 7.38 (q, *J* = 8.3 Hz, 2H), 7.29 (dd, *J* = 17.3, 8.1 Hz, 2H), 7.23 (t, *J* = 7.4 Hz, 1H), 7.18 (d, *J* = 8.0 Hz, 1H), 6.32 (d, *J* = 13.8 Hz, 1H), 6.18 (d, *J* = 13.1 Hz, 1H), 4.12 (t, *J* = 6.5 Hz, 2H), 4.07 (t, *J* = 7.4 Hz, 2H), 3.18 (q, *J* = 6.6 Hz, 8H), 3.09 (s, 1H), 2.18 (s, 2H), 2.10 (t, *J* = 7.4 Hz, 2H), 1.86 (s, 4H), 1.81 – 1.74 (m, 2H), 1.66 (d, *J* = 4.1 Hz, 11H), 1.52 (t, *J* = 7.5 Hz, 2H), 1.49 (s, 1H), 1.37 – 1.30 (m, 2H), 1.27 (s, 1H), 1.10 (q, *J* = 6.2 Hz, 10H).

HRMS (ESI): m/z: calcd for $C_{38}H_{54}N_3O^+$: 584.4211 [M^+]; found: 584.4243

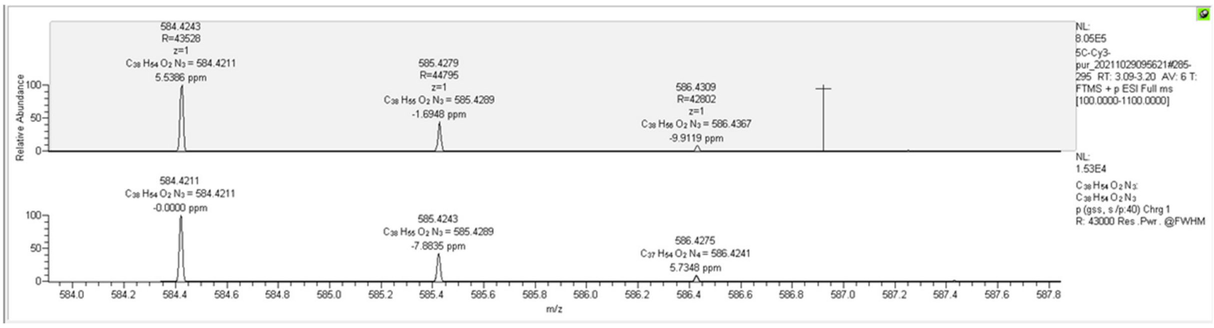

Synthesis of **BPA-Cy3**

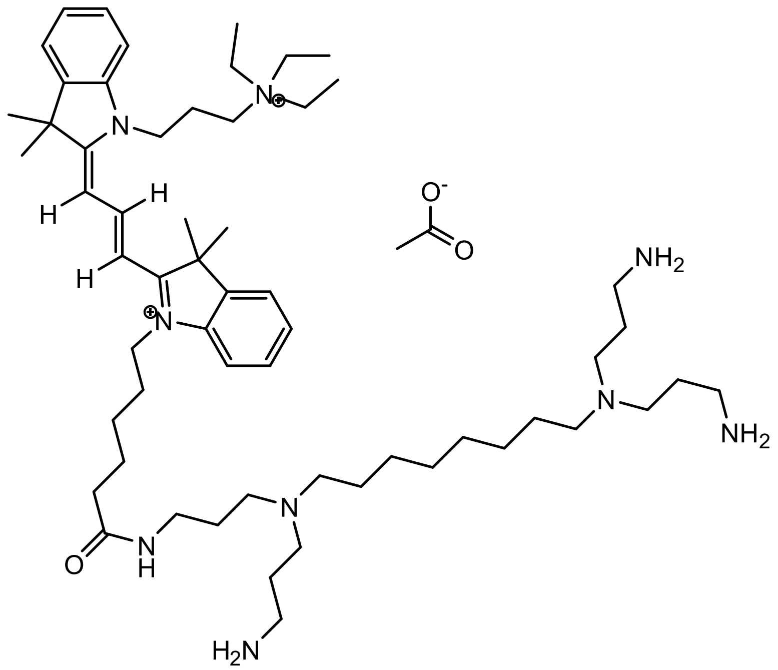

To a solution of 1-(5-carboxypentyl)-2-[(1E)-3-[(2E)-3,3-dimethyl-1-[3-(triethylazaniumyl)propyl]-2,3-dihydro-1H-indol-2-ylidene]prop-1-en-1-yl]-3,3-dimethyl-3H-indol-ium (1 eq., 96.5 mg, 0.165 mmol)) and TSTU (TSTU (2 eq., 99.2 mg, 0.329 mmol)) in DMF (4098 µL) was added triethylamine (2 eq., 33.3 mg, 45.8 µL, 0.329 mmol) and the solution was stirred at rt during 2h.. The above solution was added to a solution of bis(3-aminopropyl)({8-[bis(3-aminopropyl)amino]octyl})amine (2 eq., 122 mg, 0.329 mmol) and Na2CO3 (10 eq., 174 mg, 1.65 mmol) in water (6502 µL).The final mixture was stirred at rt for 15h.. The solvent was evaporated under reduced pressure and the residue was purified by automatic flash chromatography Biotage Isolera, (reverse phase, MeOH/H_2O with 10 % of Acetic acid) to obtain the title compound (85 mg, 75,6%) as a dark pink solid;

^{1}H NMR (500 MHz, H_2O+D_2O) δ 8.47 (t, *J* = 13.4 Hz, 1H), 7.49 (t, *J* = 8.0 Hz, 1H), 7.39 (q, *J* = 7.2 Hz, 1H), 7.30 (d, *J* = 7.9 Hz, 1H), 7.28 – 7.17 (m, 1H), 6.31 (d, *J* = 13.7 Hz, 1H), 6.19 (d, *J* = 13.1 Hz, 1H), 4.09 (d, *J* = 7.0 Hz, 1H), 3.17 (q, *J* = 7.2 Hz, 5H), 3.05 (s, 2H), 2.95 (s, 15H), 2.62 (s, 1H), 2.18 (s, 1H), 2.12 (t, *J* = 7.3 Hz, 1H), 1.91 (d, *J* = 14.0 Hz, 8H), 1.81 (s, 11H), 1.78 (s, 1H), 1.72 (s, 1H), 1.66 (d, *J* = 6.7 Hz, 6H), 1.22 (d, *J* = 11.9 Hz, 8H), 1.10 (t, *J* = 7.2 Hz, 4H).

LC/MS : m/z: calcd for $\mathbf{C_{58}H_{101}N_9O_2^{2+}}$: 469.9059 [$M^{2+}$]; found: 469.9055.

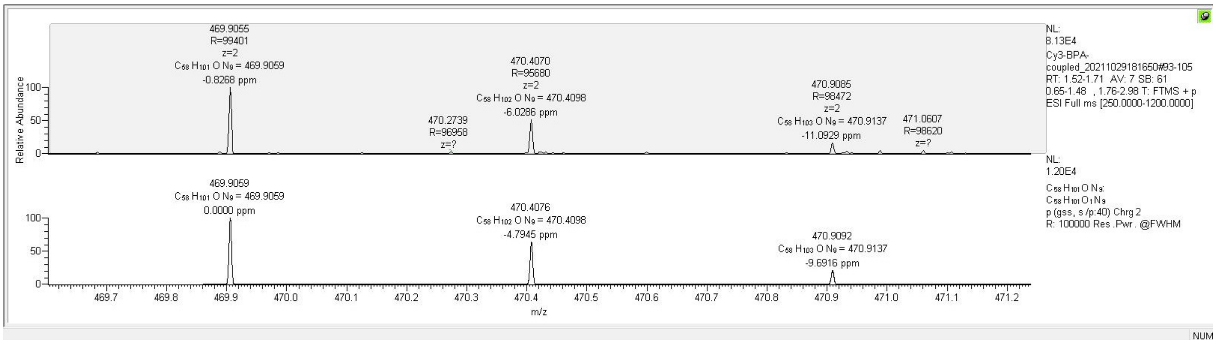

LC/MS : m/z: calcd for $\mathbf{C_{58}H_{102}N_9O_2^{2+}}$: 469.9059 [$M^{2+}$+$H^{+}$]; found: 469.9055.

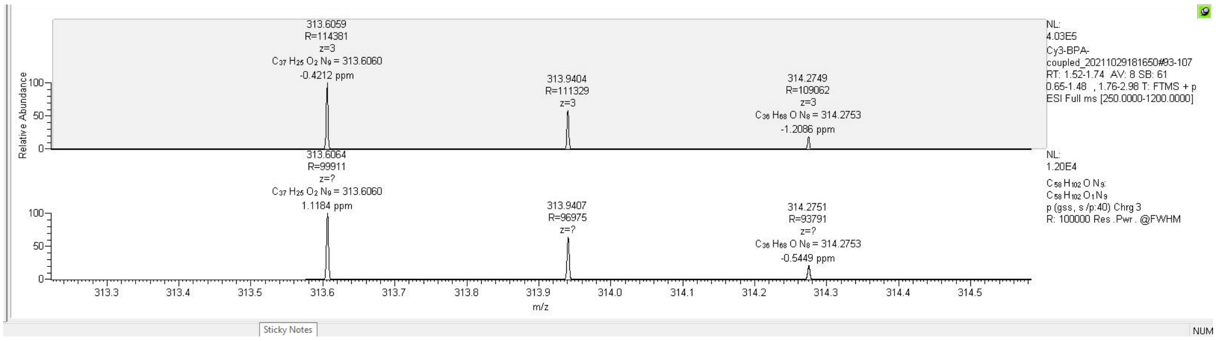

Supplement: Supplementary file 1 [file cells-10-03573-s001.zip › Supplemental Material_description synthesis of BPA-C8-Cy3.pdf]
