# Supplementary figures and images for: Effects of Dithiothreitol on Fertilization and Early Development in Sea Urchin
Source: Cells. 2021 Dec 17;10(12):3573. doi: 10.3390/cells10123573 (PMC8700669; doi:10.3390/cells10123573)

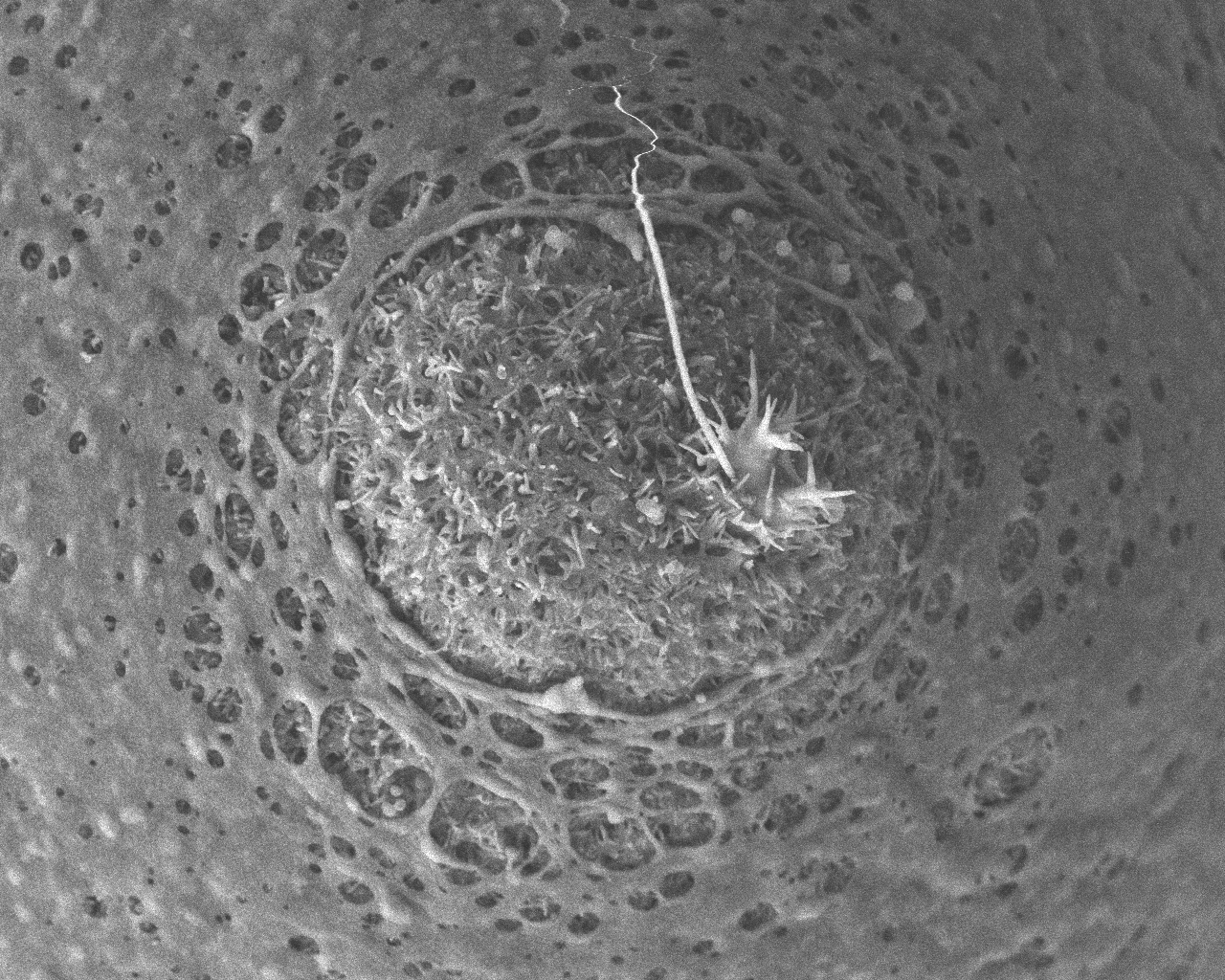

Supplement: Supplementary file 1 [file cells-10-03573-s001.zip › Figure S1.tif]
